# Why vaccines fail against *Piscirickettsiosis* in farmed salmon and trout and how to avoid it: A review

**DOI:** 10.3389/fimmu.2022.1019404

**Published:** 2022-11-17

**Authors:** Paula Valenzuela-Aviles, Débora Torrealba, Carolina Figueroa, Luis Mercado, Brian Dixon, Pablo Conejeros, José Gallardo-Matus

**Affiliations:** ^1^ Laboratorio de Genética y Genómica Aplicada, Escuela de Ciencias del Mar, Pontificia Universidad Católica de Valparaíso, Valparaíso, Chile; ^2^ Grupo de Marcadores Inmunológicos en Organismos Acuáticos, Pontificia Universidad Católica de Valparaíso, Instituto de Biología, Valparaíso, Chile; ^3^ Department of Biology, Faculty of Science, University of Waterloo, Waterloo, Canada; ^4^ Centro de Investigación y Gestión de Recursos Naturales (CIGREN), Facultad de Ciencias, Instituto de Biología, Universidad de Valparaíso, Valparaíso, Chile

**Keywords:** vaccine efficacy, coinfection, sea lice, salmonids, salmon disease, antibiotics, fish vaccine

## Abstract

Piscirickettsiosis is the most severe, persistent, and damaging disease that has affected the Chilean salmon industry since its origins in the 1980s. As a preventive strategy for this disease, different vaccines have been developed and used over the last 30 years. However, vaccinated salmon and trout frequently die in the sea cages and the use of antibiotics is still high demonstrating the low efficiency of the available vaccines. The reasons why the vaccines fail so often are still debated, but it could involve different extrinsic and intrinsic factors. Among the extrinsic factors, mainly associated with chronic stress, we can distinguish: 1) biotic including coinfection with sea lice, sealions attacks or harmful algal blooms; 2) abiotic including low oxygen or high temperature; and 3) farm-management factors including overcrowding or chemical delousing treatments. Among the intrinsic factors, we can distinguish: 1) fish-related factors including host’s genetic variability (species, population and individual), sex or age; 2) pathogen-related factors including their variability and ability to evade host immune responses; and 3) vaccine-related factors including low immunogenicity and poor matches with the circulating pathogen strain. Based on the available evidence, in order to improve the development and the efficacy of vaccines against *P. salmonis* we recommend: a) Do not perform efficacy evaluations by intraperitoneal injection of pathogens because they generate an artificial protective immune response, instead cohabitation or immersion challenges must be used; b) Evaluate the diversity of pathogen strains in the field and ensure a good antigenic match with the vaccines; c) Investigate whether host genetic diversity could be improved, e.g. through selection, in favor of better and longer responses to vaccination; d) To reduce the stressful effects at the cage level, controlling the co-infection of pathogens and avoiding fish overcrowding. To date, we do not know the immunological mechanisms by which the vaccines against *P. salmonis* may or may not generate protection. More studies are required to identify what type of response, cellular or molecular, is required to develop effective vaccines.

## 1 Introduction

One of the most devastating pathogens in the Chilean salmon aquaculture industry is *Piscirickettsia salmonis*, an intracellular bacterium that causes *Piscirickettsiosis* also known as Salmonid Rickettsial Septicemia (SRS). This systemic disease has been reported globally, affecting wild and farmed salmonids and other fish such as European seabass, white seabass, and lumpfish (1–3). *Piscirickettsiosis* has been affecting Chilean farmed salmon for nearly 40 years, causing mortalities that translate into significant economic losses, estimated at more than USD 700 million per year (4–7). The impact of this disease on the global salmon industry is significant for two reasons: 1) due to Chile having become the second largest global producer of farmed salmon, the total harvests of these salmonids during 2020 reaching 1078 million tons, which represents 26% of the global market (8), and 2) *P. salmonis* is the leading cause of death from infectious diseases of the three most widely cultivated species in Chile: Atlantic salmon (*Salmon salar*), Coho salmon (*Oncorhynchus kisutch*), and rainbow trout (*Oncorhynchus mykiss*) (9).

*Piscirickettsiosis* affects salmon when they are farmed in cages at sea, which creates many challenges for health management and increases the risk of infectious diseases being contracted (10). The challenges range from difficulties in diagnosing the causes of mortality to difficulties in applying veterinary treatments, such as administering antibiotics at the optimal doses or administering booster shots. Additionally, many biocontainment measures for diseases are impracticable in sea cages (11). In fact, the hydrodynamic connectivity between sea cages allows faster transmission across greater distances, so when an outbreak occurs in an upstream center, the disease is propelled to downstream farms through ocean currents (7, 11, 12). Therefore, controlling this bacterial disease, like others that occur in sea cages, has become a significant concern, and reducing its impact is a continuous challenge (13).

**Figure 1:**
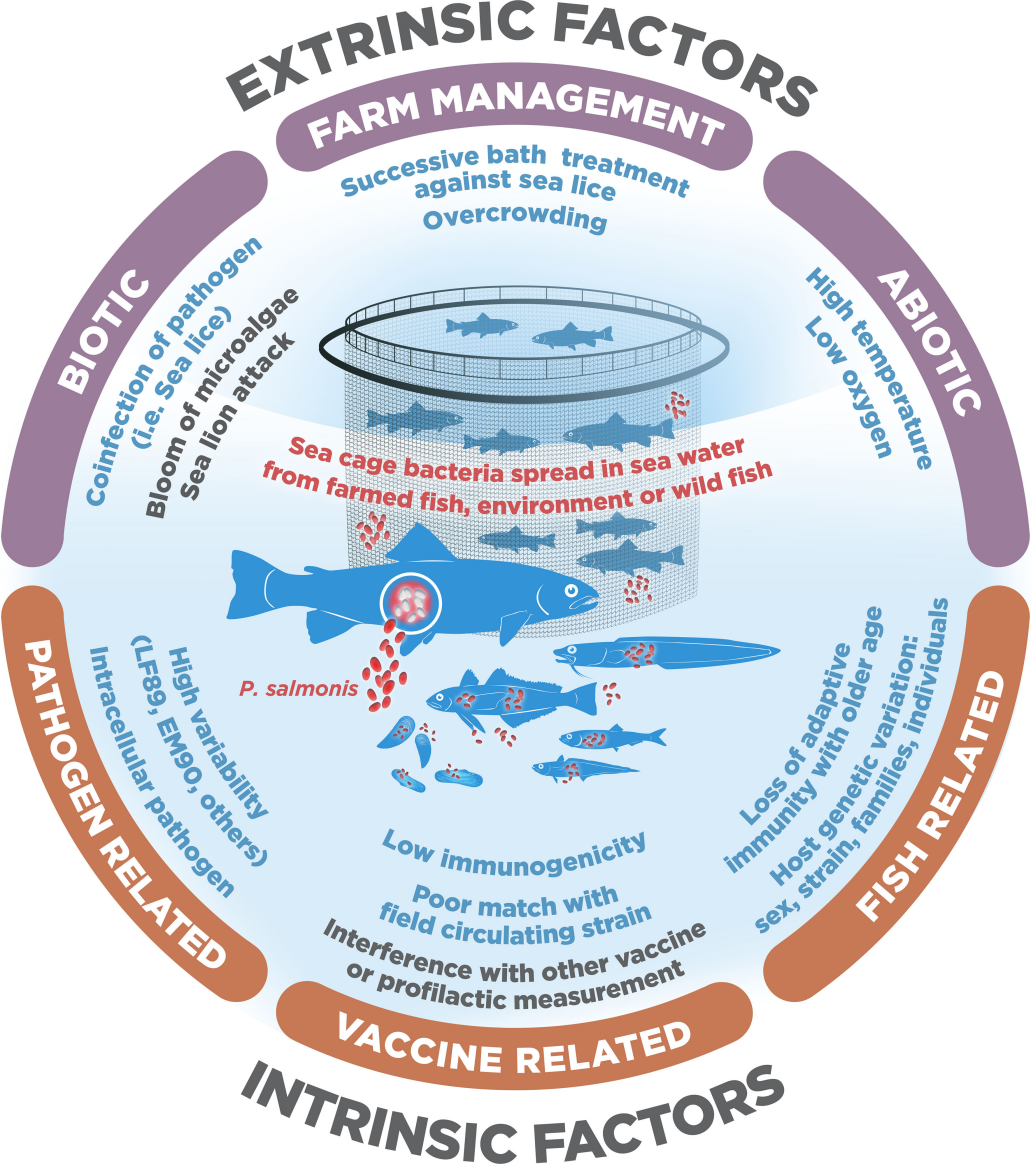
Extrinsic and intrinsic factors that decrease *Piscirickettsia salmonis* vaccine efficacy in salmon and trout Chilean farming.

There are currently several preventive strategies used to combat *Piscirickettsiosis* that the Chilean salmon industry has adopted, but the most relevant is vaccination. However, the low efficacy of the vaccines in field conditions, seen in the high mortality of vaccinated fish, continues to be one of the most critical concerns in the industry, because it induces the use of antibiotics to reduce the disease (14). The reasons for why fish vaccines fail to generate the expected efficacy in the field remain under debate (15), particularly for intracellular pathogens such as *P. salmonis*. Here, we propose that the low protection provided by vaccines against *P. salmonis* could be explained through different extrinsic and intrinsic factors that can act separately or synergistically (**Figure 1**). In figure 1, we represent the three main causes of vaccine failure for both extrinsic factors (biotic, abiotic and related to fish management), as well as intrinsic factors (fish, pathogen and related to vaccines). Further, based on the available evidence we also propose some recommendations to improve the efficacy of vaccines. Other previous bibliographic reviews can be consulted to delve into the interaction between this pathogen and its host, how it evades the immune response, the immunization strategies available, or how to treat epidemic outbreaks of this disease (12, 16–18).

## 2 Diagnosis, prevention, and treatment strategies for the control of *Piscirickettsiosis*

From the first severe Chilean outbreak in 1989 that killed more than 1 million fish, different health measures have been proposed to prevent and treat *Piscirickettsiosis* (19). These include timely diagnosis, reducing stress, administering vaccinations or immunostimulants, applying antibiotics, and, more recently, selectively breeding resistant fish (12, 20, 21). However, it was not until 2012 that a specific national surveillance and control program was implemented by the Chilean National Service for Fisheries and Aquaculture (Servicio Nacional de Pesca y Acuicultura, SERNAPESCA) (22). This program has established two specific objectives: 1) the early detection and monitoring of SRS cases and 2) the application of timely and gradual control measures in early and advanced cases of *Piscirickettsiosis*. This program allows to take a global look to the impact of this disease and the measures applied for its prevention and control.

**Figure 2:**
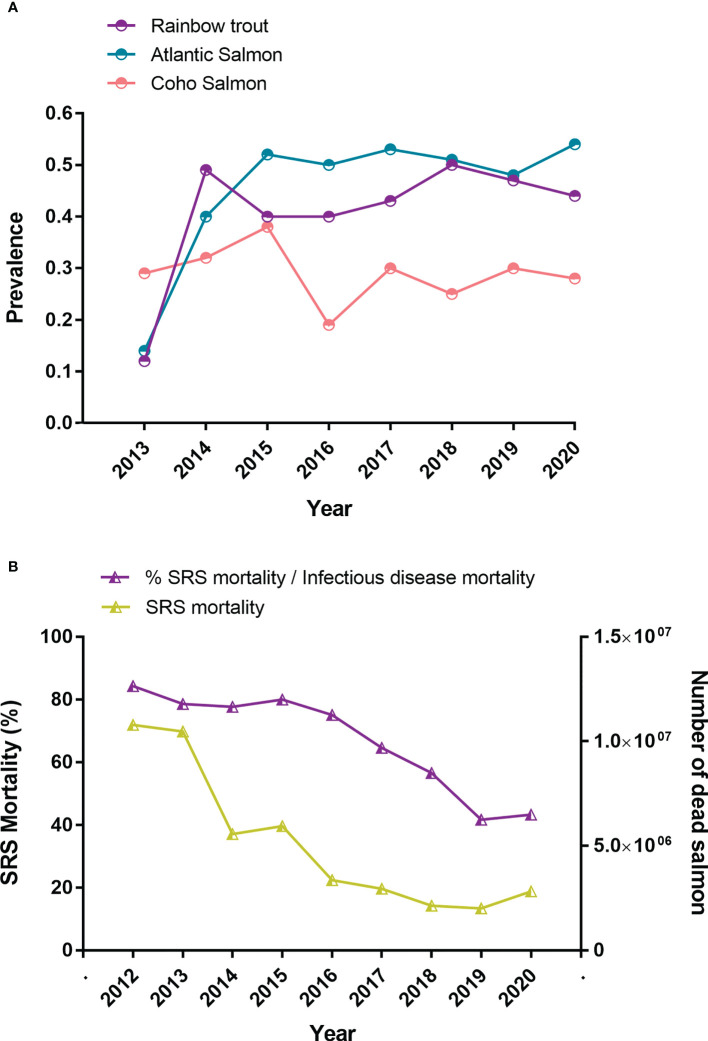
Impact of *Piscirickettsiosis* in farmed salmoninds in Chile. **(A)** Prevalence of *Piscirickettsia salmonis* in marine centers from 2013 until 2020. **(B)** Mortality due to *P. salmonis* in relation to the total mortality due to infectious causes from 2012 until 2020 (source: National Fisheries Services of Chile, 2022). Note that prevalence monitoring became more robust in 2014 after a year of its implementation in Atlantic salmon and rainbow trout.

The SERNAPESCA program requires the active surveillance of diagnostics and mortalities through a network of selected laboratories and certified procedures (22). The diagnosis is carried out one month before transfering to the sea, once the fish enter the marine centers, every two months after entry, or in the event of an increase in mortality that can be attributed to *P. salmonis* through symptoms (22). The SRS diagnosis can be demonstrated *via* different procedures, including GRAM and GIEMSA stains, histopathological examinations, isolated tissue cultures, direct and indirect immunofluorescence (DFAT and IFAT, respectively), immunoperoxidase staining, and enzyme-linked immunosorbent assays (ELISAs) (23–25), however, the polymerase chain reaction (PCR) test is the main method used in Chile, due to its sensitivity and specificity (26). Each diagnostic laboratory must report weekly their positive and negative results to the authorities, thus establishing a registry that can be consulted through an Information delivery system protected by the Chilean transparency law (22). The tissues usually chosen for diagnosis and isolation include kidney, spleen, liver, blood, gills, and brain (23–25). The surveillance program is now fully mature and allows adequate monitoring of the disease throughout Chile, even in the most remote regions. Analyses of the positive and negative cases of *P. salmonis* have established that the prevalence remained relatively constant between 2013 and 2020 and that all salmonid species are affected (**Figure 2A**). In Chile, between 2012 and 2020, around 46 million salmon and trout have died from *P. salmonis* (**Figure 2B** and **Table 1**), which represents about 9% of all deaths recorded in the industry during this period. However, the importance of this pathogen has decreased, as a proportion of deaths from infectious causes, from 84% in 2012 to 43% in 2020 (**Figure 2B** and **Table 1**).

**Table 1:** Mortality (number of fish and %) of farmed salmon and trout in Chile between 2012 and 2020.

Year	Total mortality (n)	Total infectious disease mortality (n)	SRS mortality (n)	Ratio SRS mortality/Total mortality	SRS mortality/Infectious disease mortality
2012	68635870	12791539	10789071	15.7%	84.3%
2013	81022577	13319934	10464566	12.9%	78.3%
2014	54012183	7158143	5561632	10.3%	77.7%
2015	56055495	7430929	5942986	10.6%	80.8%
2016	95399494	4475998	3361588	3.5%	75.1%
2017	36879495	4567139	2951765	8.0%	64.6%
2018	39087972	3769459	2134474	5.5%	56.6%
2019	37500780	4832404	2014123	5.4%	41.7%
2020	50558686	6520342	2825343	5.6%	43.3%
Total	519152552	64865887	46045548	8.9%	71.0%

**Figure 3:**
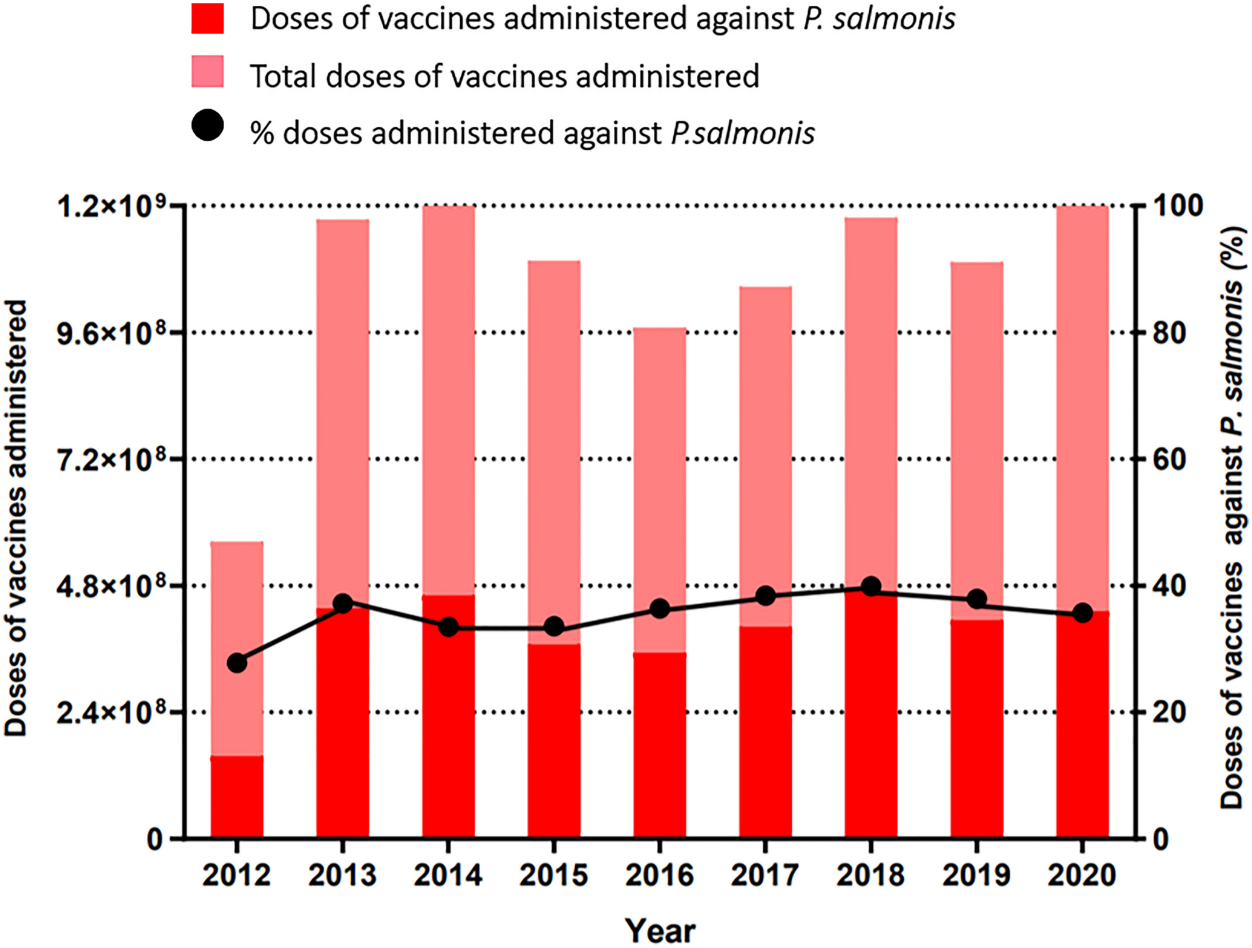
Doses of vaccines administrated in Chilean salmon aquaculture (light red) and vaccines administrated to combat *Piscirickettsia salmonis* (dark red) from 2012 until 2020. Source: National Fisheries Services of Chile, 2022.

Vaccination is the main control strategy used in aquaculture for the prevention of infectious outbreaks (27), but when this fails, antibiotics and other control measures are required. There is a great variety of vaccine types and an even wider diversity of application strategies. There are about 34 commercial vaccines licensed for use against *Piscirickettsiosis* by the Chilean Agricultural and Livestock Service (Servicio Agrícola y Ganadero, SAG) (28), which vary in the following aspects: 1) composition: monovalent or pentavalent; 2) principal constituents, such as bacterin, subunits, or live attenuated bacteria; 3) the strain of *P. salmonis*; and 4) the adjuvants used in the final preparation. Approximately 1078 million doses of vaccines were administered in Chilean salmon aquaculture in the past five years; almost 36% of these vaccines were used to prevent SRS (**Figure 3**). Differences in the efficacies of some commercial vaccines have been reported in field studies (7, 29, 30). The largest study comparing the efficacy of vaccines in the field was carried out by Happold et al. (2020), which evaluated 4798 cage-level production cycles between 2004 and 2018. They showed that in Atlantic salmon and rainbow trout, some freshwater vaccination regimens were better than others in controlling *Piscirickettsiosis*. In fact, the best vaccine evaluated in Atlantic salmon reduced mortality due to *P. salmonis* by 22% when compared to a reference vaccination regimen (25). However, in others, mortality rates were 2.6 times higher than in the reference regimen.

**Figure 4:**
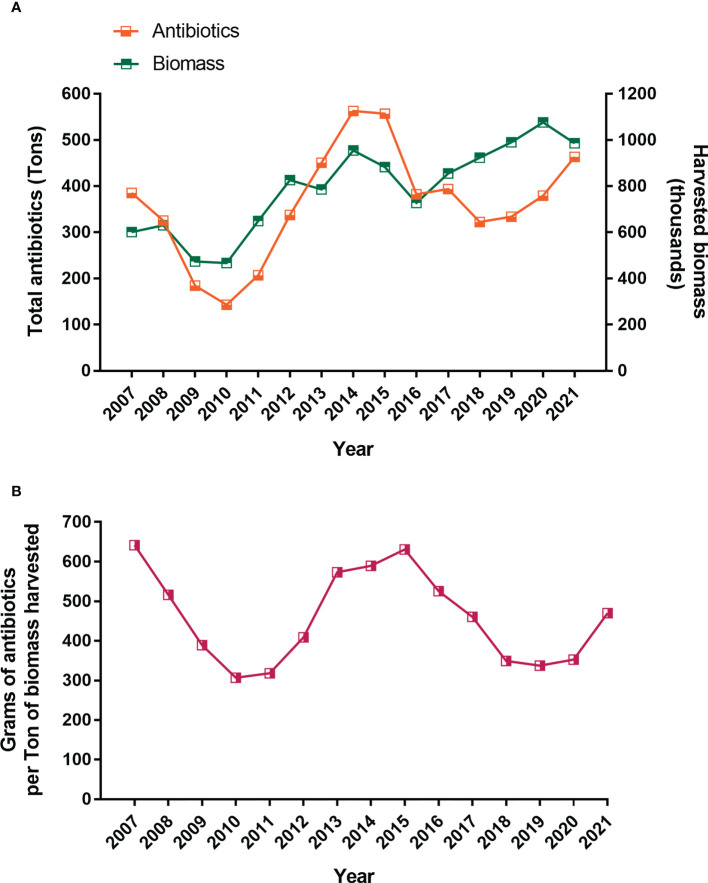
Antibiotics used and harvested biomass of farmed salmon and trout in Chile from 2007 until 2021 **(A)**. Ratio of antibiotics harvested biomass **(B)**. Source: National Fisheries Services of Chile, 2022.

Despite the large number of doses administered, three key parameters remain high in the last 10-15 years: a) prevalence of *P. salmonis*, b) mortality associated to *P. salmonis* and high use of antibiotics has been observed (**Figures 2**, **3**). According to the official statistics from the last nine years, about 413.5 tons of antimicrobial compounds were used in Chilean salmon farms in 2021 (31), and almost 90% of the total antibiotics applied used to treat *Piscirickettsiosis* (**Supplementary Table 1**). Surprisingly, the trend from the last 15 years shows a cyclical pattern in the use of antibiotics (**Figure 4**), which cannot be predicted either by prevalence (**Figure 1A**) or by the constant use of vaccines (**Figure 3**). Thus, some authors suggest that vaccines are failing in their protective role and that the control of SRS is highly dependent on antimicrobials for combating outbreaks and limiting the pathogen’s spread (32, 33). On contrary, since they confront mainly viral diseases, Norwayan aquaculture used only 223 kilograms of antibiotics for 1.4 million tons of salmonids in 2020 (34), which is 0.059% of the antibiotics used in Chilean aquaculture for the same year. Although antibiotics reduce the mortality induced by *Piscirickettsiosis*, there is significant concern due to the potential development of antimicrobial resistance and the accumulation of antibiotic residues in the environment (35, 36).

## 3 Why vaccines fail against Piscirickettsiosis?

### 3.1 Extrinsic factors

Within the extrinsic causes, we include all those factors that depend on the management conditions of the fish or the environment in which they are farmed, whether they can be controlled or not. Extrinsic factors for which a detrimental effect on the efficacy of vaccines has been shown or suggested usually are associated with different types of chronic stressors. It is well known that chronic stressors suppress the immune response and increase pathogen infection in fish (37). In contrast, it is still poorly understood how short-term acute stressors might influence the efficacy of vaccines. Thus, we divided the extrinsic factors into: 1) abiotic factors, such as high temperatures or low oxygen in sea cages; 2) biotic factors, such as coinfections with sea lice or sealion attacks; and 3) farm-management factors, such as fish overcrowding (**Figure 1**).

#### 3.1.1 Abiotic factors

Several studies have demonstrated that *P. salmonis* survives in seawater (10, 38, 39), where horizontal aquatic transmission of the pathogen in the absence of parasite vectors has been proposed (11, 38). Therefore, the transmission of *P. salmonis* due to hydrodynamic connectivity between farms contributes to the emergence and progression of *Piscirickettsiosis*, as farms are generally significantly interconnected through ocean currents (11, 40–42). Thus, the seawater temperature and salinity have been described to have a relevant influence on the pathogen’s risk of transmission and prevalence. Water temperatures ranging from 9°C to 16°C, present during the fall and spring, and at a salinity > 26 practical salinity units (PSUs), increase risk of *Piscirickettsiosis* (11, 41, 42). However, the mechanisms by which the temperature and salinity influence the disease dynamics remain unclear, although higher temperatures are associated with increased host and pathogen metabolic activity (43). Moreover, *P. salmonis* outbreaks often appear after a period of high variation in water conditions, such as after storms and low-oxygen conditions (controlled by ocean-current velocities and solubility) (11, 32).

#### 3.1.2 Biotic factors

**Figure 5:**
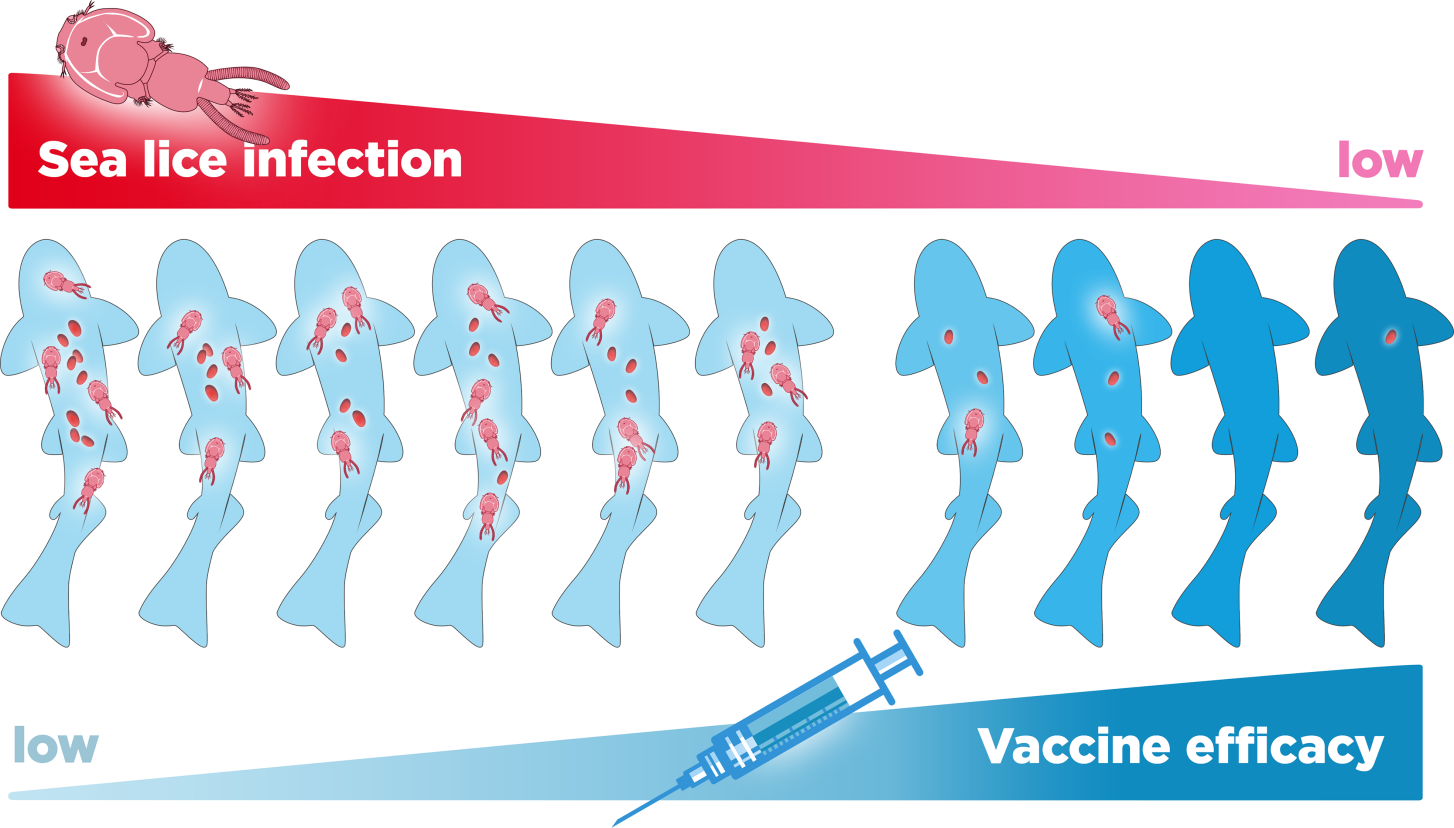
Relationship between level of infestation with the sea lice *Caligus rogercresse*yi and the loss of efficacy of vaccines against *Piscirickettsia salmonis* in farmed salmon.

Coinfections with the sea louse *Caligus rogercresseyi* have been observed to occur naturally in salmon farms and have been associated with detrimental effects on the resistance to *P. salmonis* in both unvaccinated and vaccinated fish (44, 45). Sea lice are among the most common parasites for the global salmon industry, with a prevalence of up to 100% in some farms (46, 47). Moreover, in unvaccinated fish, the infestation of salmonids with sea lice can cause lethal or sublethal effects such as stress, loss of appetite, decreased growth, skin damage, depression of the immune system, and flesh quality detriment (32, 45, 48–50). Nevertheless, it has been established that the sea louse maintains only a transient association that does not last more than one hour, so it is not considered a biological or mechanical vector of *P. salmonis* (51). On the other hand, in vaccinated fish, it has been shown that the sea louse *C. rogercresseyi* can override the protective effects of a commercial *P. salmonis* vaccine in Atlantic salmon (44) (**Figure 5**). Under laboratory conditions, coinfection with these parasites reduces the survival and growth of vaccinated fish, increasing the bacterial load and pathological signs of disease, leading to acute SRS infections (44). Similarly, in field conditions where a high percentage of fish are vaccinated against *P. salmonis*, it has been observed that a high prevalence of adult sea lice is significantly associated with *Piscirickettsiosis* cumulative mortality, suggesting that the two diseases have a synergistic relationship (50). Thus, sea-lice coinfection with other pathogens could partly explain the low vaccine efficacy in field conditions. Coinfection highlights the need for in-depth studies on the effects of commercial vaccines in more diverse conditions, i.e. considering the combined effect of the different pathogens that are regularly found in the cages of aquaculture centers.

Two other biotic factors have been linked to chronic stress and mortality due to *Piscirickettsiosis in* salmon farms: sealions and harmful algal blooms. Sealion attacks and the predation of salmon have also been proposed as strong stressors that increase the risk of *P. salmonis* outbreaks in salmon farms (52). The sealion *Otaria flavescens* is abundant in the waters of southern Chile, and its predation on farmed salmon has been reported (53). Predation usually occurs due to poorly implemented isolation structures (i.e., meshes for excluding sealions). Farmers report an increase in *P. salmonis* mortality during or after sea lion attacks, particularly when sea lions attack cages constantly (53). Therefore, companies must devote significant resources to the prevention of sealion attacks, not only to reduce predation but also to avoid *P. salmonis* outbreaks. Additionally, farmed salmon are extremely vulnerable to harmful algal blooms (54). There are a variety of physiological mechanisms that can act individually or in combination to cause stressful effects on fish, such as 1) physical damage to the gills; 2) ichthyotoxins, metabolites that are toxic to fish that can be produced by some species of algae; 3) hypoxia in the blood due to reduced ambient oxygen; and 4) gill injuries and gas-bubble trauma due to extreme oxygen supersaturation caused by algal photosynthesis (54–56). The most effective mitigation strategy for the industry to reduce the harmful effects of algal blooms is food retention (55). Nevertheless, over extended periods, this can increase the physiological stress in the fish and susceptibility to chronic infections such as bacterial kidney disease (BKD) and SRS (54, 57).

#### 3.1.3 Farm-management factors

At least two management practices have been strongly associated with increased outbreaks of *P. salmonis*: the number of bath treatments used to combat sea lice and overcrowding. As previously mentioned, sea lice have been associated with increased mortality in vaccinated fish. If the load of sea lice is high, the number of antiparasite dip treatments carried out by the farming centers increases (52), and higher rates of bathing treatments are associated with a higher risk of mortality attributed to SRS (7, 58), due to the acute stress generated by the bathing process. Another factor of farm management is the high number of fish per cage. Salmon overcrowding intensifies friction and fish aggressiveness which causes skin damage (7).

Other management practices that may contribute to infection include the movement of diseased fish between different farming areas, the resting time between the beginning and end of the fattening stage, and the *in situ* cleaning of nets. Aquaculture often involves the movement of large numbers of animals between farms, such as salmon from hatcheries move to freshwater production sites before being moved to marine grow-out sites (59, 60). The transfer of animals can cause a network of interactions between fish groups from different farms, increasing the probability of disease spreading if the site of origin is infected; therefore, coordinated cleaning between transfers reduces the transmission of the disease (59). On the other hand, the risk of developing SRS is also proportional to the time salmonids spend in seawater (42). On average, there was an increase in the possibility of SRS being reported after the fifth month in marine centers, with the rainbow trout and coho salmon species showing the greatest possibility of SRS reporting throughout the end of the production cycle (41). Additionally, the reduced resting time and occurrence of neighboring infected farms have been associated with high risk of SRS (42). Thus, a prolonged resting time without hosts in a farm is a good health strategy, particularly because *P. salmonis* cannot survive or replicate in salt water over a prolonged period of time (10). Recently, it has been found that the fouling of the cages may increase the stress in the fish due to reductions in the ventilation and the availability of oxygen (52). Thus, good cleaning of encrustations in fishing nets, the use of antifouling systems, and the properly coordination of resting time are essential factors to be considered for good management.

### 3.2 Intrinsic factors

As intrinsic factors, we include all those factors directly related to the interaction between the fish, the *P. salmonis* pathogen, and the vaccines. Thus, we divided the intrinsic factors into 1) fish-related, including their genetics, sex or age; 2) pathogen-related, including their variability and ability to evade host immune responses; and 3) vaccine-related, including low immunogenicity and poor matches with the circulating pathogen strain (**Figure 1**).

#### 3.2.1 Fish-related factors

**Figure 6:**
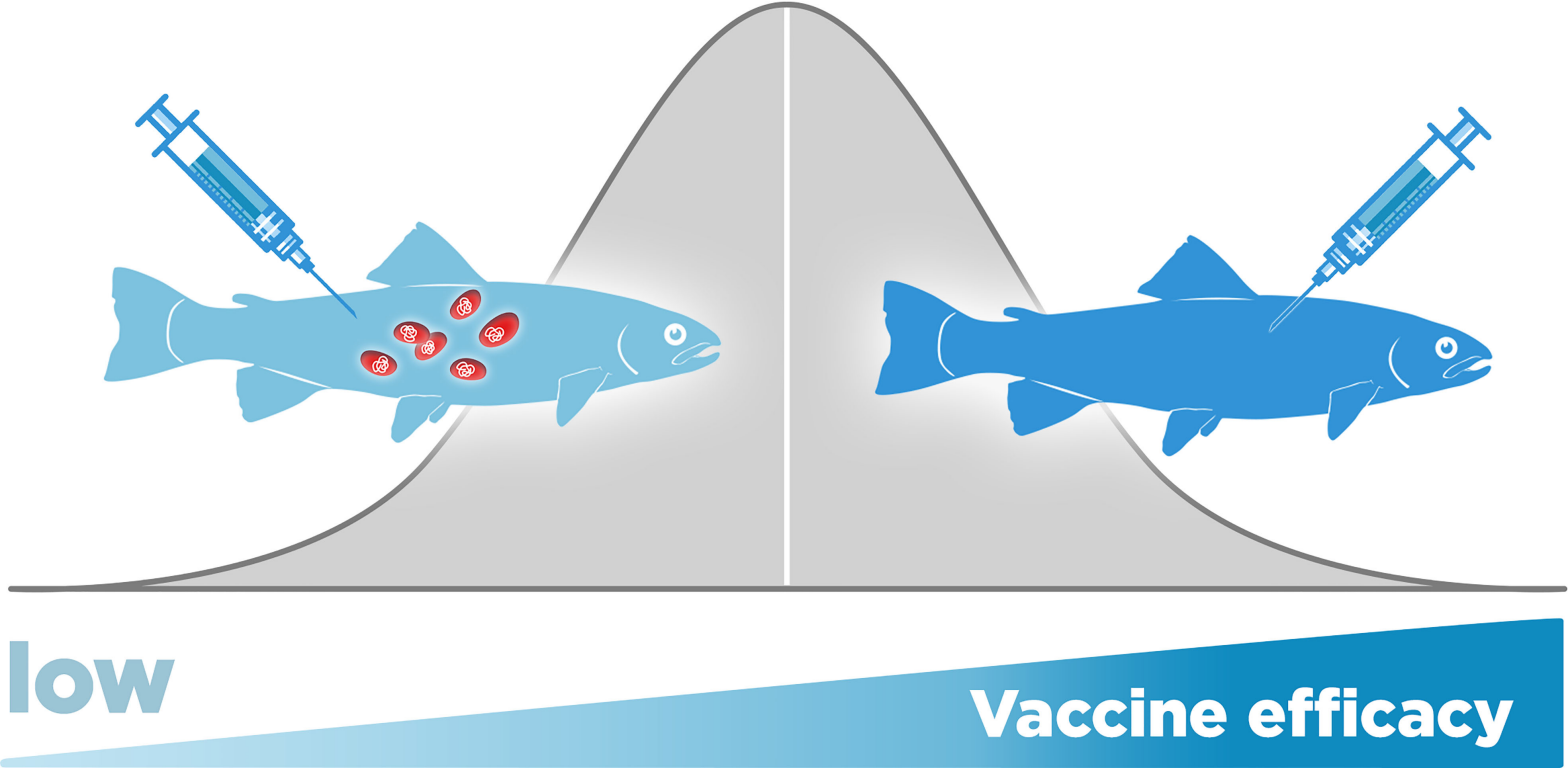
*Piscirickettsia salmonis* vaccine efficacy in salmonids is influenced by host genetic variation. While in some fish, the protection added by vaccination increased the survival and reduce the bacterial load (right fish) in other, there is no added protection by vaccination (left fish).

Even under optimal conditions, some fish may not be protected after vaccination, which may be a direct consequence of the host’s genetic variability (20). Natural variation in the resistance against *P. salmonis* in unvaccinated fish has been demonstrated between species (61), as well as within species, including Atlantic salmon, rainbow trout, and Coho salmon (62, 63). Recently, it has been described that vaccine efficacy is also influenced by heritable genetic variation among the hosts, and, as above, differences both between and within species have been observed (20). Results have shown that there are fish families in which the vaccine does not protect against *P. salmonis* and other family groups in which the vaccine-mediated protection is high (20). In **Figure 6**, we represent this phenomenon, while in some fish, the protection added by vaccination increased the survival and reduce the bacterial load (right fish) in other, there is no added protection by vaccination (left fish). Currently, the genes that control this genetic variation in vaccinated fish are unknown. Moreover, it is hypothesized that the genes involved in natural resistance in unvaccinated fish may not be the same genes involved in resistance in vaccinated fish (20).

**Figure 7:**
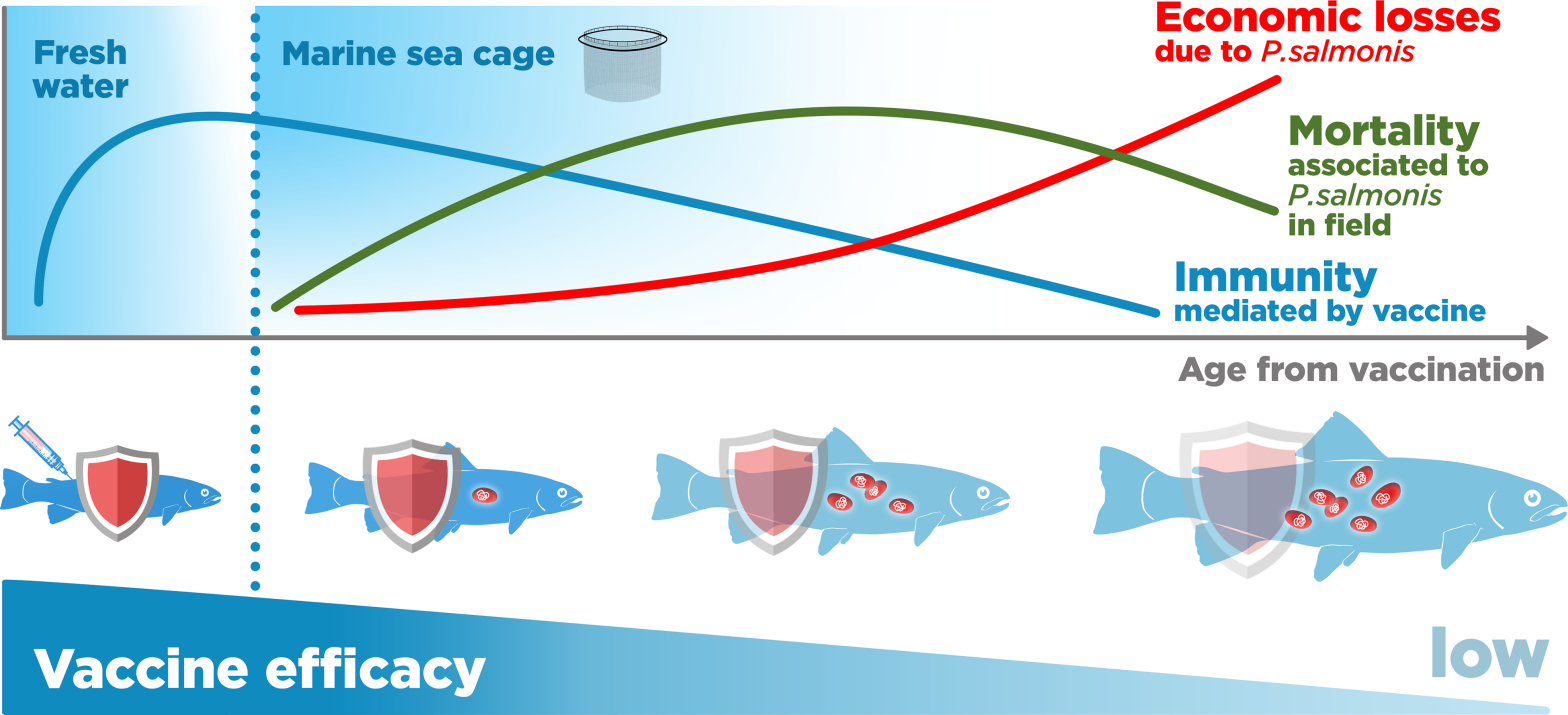
*Piscirickettsia salmonis* vaccination-mediated immunity and its relationship with mortality and economic losses in salmon farming in sea cages.

Although studies on the *P. salmonis* vaccines’ effectiveness in the field are limited, there is some evidence and consensus in the industry that the long-term protection conferred by *P. salmonis* vaccination is low (16, 32, 64). Loss of protection after an initial period of effectiveness may occur in some fish and may be related to a decrease in the circulating anti-*P. salmonis* antibodies (antigen-specific IgM) with age (65) (**Figure 7**). Tobar et al. reported that fish immunized with commercial vaccines presented maximum antibody (IgM) concentrations between 600 and 800 degree-days post-vaccination (dpv) (60 to 80 days at 10°C) (65). After this, they decrease drastically, reaching pre-vaccination levels, near 1800-1900 degrees dpv. Thus, Atlantic salmon may not achieve protection when they reach harvesting size at 3000 to 6000 degrees dpv (12, 65) (**Figure 7**). A practical measure by which to prevent this decline could be the application of oral supplements, as suggested by Tobar and collaborators, but there is no consensus that this strategy is effective in the field (29, 65).

In addition to the increase in antibodies, other defense mechanisms for combating *P. salmonis* have been described for the host. For instance, iron deprivation in unvaccinated Atlantic salmon limits the bacteria’s access to this essential nutrient for their growth (66, 67). Moreover, reactive oxygen intermediates generate respiratory bursts and interact with possible resistance markers such as interleukin receptors and fucosyltransferase genes (21, 68). Extracellular traps (ETs) act by trapping *P. salmonis* through DNA networks, histones, and antimicrobial peptides (69).

The vaccine’s immune response is generally characterized by the number of antibodies raised. However, other actors should be triggered to activate a relevant immune response that avoids or control the infection by Piscirickettsia in salmonids. Vargas and collaborators study reports that the immunization of Atlantic salmon with a live commercial vaccine induces a short-term upregulation of the cellular-mediated immune response at 5 dpv modulated by the upregulation of *ifnα*, *ifnγ*, and the *cd4* and *cd8α*, but a decrease response at 15 and 45 dpv in field conditions (30). Another study evaluated an inactivated whole-cell bacterin of *P. salmonis* in controlled conditions in Atlantic salmon (70); the experimental vaccine induced upregulation of mediators of innate immunity and proinflammatory cytokines genes that decrease over time, such as *ifnγ*, *tnfα*, *il-1β*, *il-10*, *il-12β*, and the upregulation of cell-mediated and humoral immunity genes, such as *mhcI*, *mhcII* and *cd4* (70). However, the vaccine produced the downregulation of *cd8β* and *igm* after 7 dpv supported the CD4+ T-cell response but did not induce an immune response mediated by CD8+ T cells or a humoral response (70). Moreover, the evaluation of experimental vaccines based on *P. salmonis* recombinant proteins and proteoliposome reports the upregulation of genes related to innate (14, 71), and cell-mediated immunity adaptive immune response activation (14). The immune response activated by commercial and experimental vaccines has been described and reviewed extensively elsewhere (12, 16, 18).

The development of efficient vaccines against *P. salmonis* has been hampered for 30 years due to poor knowledge regarding the pathogen–host interaction, the limitations of fish memory cells, and the mechanisms used by intracellular bacteria to avoid CD8+-cell activation (18, 71, 72). Moreover, there is no evidence indicating that vaccines activate the cellular-mediated immune responses necessary to control intracellular pathogens (18, 73). More in-depth knowledge of the immune system of salmonids and a better understanding of the immunological mechanisms involved in the host–pathogen interaction and the genes related to genetic variation in vaccinated fish are essential for the correct use and development of more efficient vaccines against *P. salmonis*.

#### 3.2.2 Pathogen-related factors

*P. salmonis* is a bacterium within the class of γ-proteobacteria (74),in the order Thiotrichales and family Piscirickettsiaceae. *Piscirickettsia* was first isolated in 1989 from Coho salmon (*Oncorhynchus kisutch*), but it was not until 1992 that the name *P. salmonis* was assigned (75). The first draft of the sequenced genome of *P. salmonis* strain LF-89 (VR-1361) was published in 2013 (76). However, it was only in 2015 that its genome was sequenced entirely, it consisted of a circular chromosome of 3.2 Mb and three plasmids (77). More recently, the number of plasmid per genome range from one to seven (Mode = four), ranging in size from 9 to 251 kb (78). A pangenomic analysis of 19 strains of *P. salmonis* resulted in their division into two genogroups, LF-89 and EM-90, based on their geographic distributions, antimicrobial susceptibilities, growth kinetics, and host specificities (79). However, three genogroups are now recognized globally, including one of Canadian and Norwegian origin (78). They differ mainly in the size of the genome, in the number of genes and in the number of plasmids (**Table 2**). The EM-90 genogroup contains specific virulence factors related to adherence, colonization and invasion, and endotoxins that make them more virulent than the LF-89 genogroup (70, 79–81). Moreover, EM-90 is widely disseminated and is responsible for a significant portion of the SRS cases in Atlantic salmon in Chilean marine farms (82), whereas LF-89 was isolated from only three breeding species in Chilean farms (82). On the other hand, the specificity of pathogens for a host does not imply that the pathogens cannot not cross to other hosts (82, 83). Indeed, the most recent analysis of 73 complete genomes revealed evidence for co-occurrence of LF and EM genogroup strains within the same individual host (78).

**Table 2:** Genetic characteristics of the 3 known genogroups of *P. salmonis*. Adapted from Schober (78).

Year	LF	EM	NC (Norway - Canada)
Genome size [Mb]	3.42 - 3.60	3.25 - 3.71	3.77 - 4.15
Genes in genome	3,549 - 3,696	3,371 - 3,956	4,125 - 4,955
Number of plasmids	3 - 5	1 - 5	5-7

*P. salmonis* is a facultative intracellular bacterium that can replicate both within the host and extracellularly (80). Despite significant advances in elucidating the mechanisms of infection of intracellular pathogens such as *P. salmonis*, it has not yet been possible to establish how this pathogen can evade and control the host’s immune response (18). The bacterium can be found inside cells in large vacuoles within the host cell’s cytoplasm, displacing the nucleus to one side, allowing it to reside and proliferate (81, 84, 85). *Piscirickettsia* can modulate the immune system by inhibiting the expression of the driver IL-12 cytokine associated with cellular innate immune response and a bridge signal with adaptive immunity; and *P. salmonis* can induce the over-expression of IL-10 an anti-inflammatory cytokine, in the early stages of infection (86). Thus, *Piscirickettsia* could promote proliferation through the inhibition of macrophages, apoptosis, and the synthesis of cytokines in salmonids, preventing the induction of an inflammatory response and promoting their own survival within host cells (86–88). Additionally, it has been proven that *P. salmonis* is capable of modulating CD8+ lymphocytes and altering antigen processing and presentation as a mechanism for evading cell-mediated responses (18, 89). Furthermore, it has been demonstrated that this pathogen promotes an antibody-mediated response, activating CD4+ lymphocyte mediated responses (89). These antibody responses are insufficient to counteract *P. salmonis* when it is invading the host, as demonstrated by the low efficiency of commercial vaccines under field conditions. This evidence adds to the fact that *P. salmonis* inhibits vesicular trafficking through disrupting the organization of actin and microtubules in the cytoskeleton (81). Furthermore, it prevents fusion with vesicles containing the antimicrobial peptide hepcidin, a component of the innate immune system necessary for eliminating pathogens (86).

It has been shown that *P. salmonis*, like other intracellular bacteria, has secretion systems to deliver multiple proteins, called effectors, which participate in trafficking the host membrane to establish an intracellular replication niche (90). One of the best-characterized systems in *P. salmonis* is the type IV secretion system Dot/Icm (90). The expression of the type IV secretion system increases during infection, leading to the inhibition of phagosome–lysosome fusion and thus favoring the replication of intracellular bacteria in the host, thereby increasing infectivity (90–92).

Another pathogenic mechanism is the formation of outer membrane vesicles (OMVs). *P. salmonis* constitutively releases OMVs into the host cell, which have been implicated in the delivery of virulence factors (93). These virulence factors play diverse roles in bacterial pathogenesis, including invasion, adherence, antibiotic resistance, host cell damage, immunomodulation, and biofilm formation (93).

Biofilms are a mechanism used by bacterial clusters to survive and persist in the presence of stressors such as antimicrobials and disinfectants (94). P*. salmonis* produces biofilms in response to stressful environmental stimuli and can thus survive within marine environments (95). Biofilm formation has recently been hypothesized to generate pathogen tolerance to various biologically active molecules present in the fish skin mucosa (96). The latter is involved in the modulation exerted as a defense mechanism to inhibit the innate immune response (96, 97). Therefore, the biofilm produced by *P. salmonis* can be considered a relevant virulence factor.

Another essential virulence factor that has been characterized is the presence of proteolytic enzymes, which actively participate in the invasion and proliferation of the bacterium within the host (98). At the transcript level, the expression of the *hlyA* gene has been identified, which would indicate that *P. salmonis* can secrete leukotoxins as virulence factors (98). This could explain several of the signs of *Piscirickettsiosis*, such as the anemia and hemorrhages observed in multiple visceral organs (32).

In addition, it has been suggested that *P. salmonis* may be capable of inhibiting the translation machinery of proteins synthesized by immune cells as a defense mechanism (66). All of the above should be considered in addition to the recent evidence that *P. salmonis* and host genes are involved in the biosynthesis/degradation of amino acids (99). Thus, it is suggested that there could be a transcriptional modulation of the expression of the amino acids leucine, valine, and isoleucine by *P. salmonis* since the pathogen would take up these amino acids as a source of carbon and energy (99).

Therefore, the development of metabolic plasticity in resource-limited environments could be one of the crucial mechanisms for the survival of the bacterium and the development of virulence (99). *Piscirickettsia*’s survival and adaptation are directly related to the transfer of information through mobile genetic elements (MGEs) (80). MGEs may benefit the bacteria’s traits such as their intake or degradation of nutrients, their resistance to metals and antibiotics, and their virulence (80, 100). Moreover, the sequencing of *P. salmonis* revealed that it is able to develop iron-acquisition mechanisms controlled by the availability of iron in the medium (66). Genes involved in iron acquisition from the medium could act as a virulence factor by detecting iron fluctuations and adaptively responding to iron deficiency and excess (66). Regulation of the acquisition and detoxification systems in the bacterium has been demonstrated (66).

All of the above converges on the hypothesis that *P. salmonis* selectively modulates the signaling of synergistic alternative pathways of the host’s innate immune response, allowing it to infect the host and maintain itself inside the cell.

#### 3.2.3 Vaccine-related factors

Contrary to the detailed information available from the public records regarding diagnosis, mortality, or the doses of vaccines/antibiotics provided by SERNAPESCA, little information is available to assess the efficacy and immunity conferred by commercial vaccines in Chile. However, some small pieces of information could be obtained from the information leaflet regarding 28/34 vaccines with provisional registration, as detailed in **Supplementary Table 2**. Of the 28 vaccines, 85.7% (24/28) are claimed to prevent *Piscirickettsiosis*, while only 10.7% (3/28) are declared able to reduce the mortality, clinical signs, or damage associated with this disease. In terms of the immunological properties, 46.4% (13/28) are claimed to be able to stimulate active immunity against *Piscirickettsiosis*, while 53.5% (15/28) do not have such claims in their leaflets. On the other hand, only 21.4% (6/28) clearly established the onset of protective immunity with a minimum of 456 accumulated thermal units (ATUs) and a maximum of 600 ATUs after vaccination. None of the vaccines with provisional registration that were analyzed provided information regarding the duration of protective immunity. A similar phenomenon can be observed for the relative percent survival (RPS): only five of 28 pharmaceutical companies provide information on the RPS_60_, which are greater than 80% in all cases. Nonetheless, we can assume that all the others must have reached the minimum values to obtain their provisional registrations. Regarding immunogenic compatibility, 57.15% (16/28) have no available information regarding the safety and efficacy of concomitant use with other pharmaceutical products. Moreover, 42.8% (12/28) do not have any information regarding compatibility (**Supplementary Table 2**). Finally, most of the vaccines are injectable 94% (31/34), 2 are administered orally, and only one is by immersion. Analysis of how these licensed vaccines are used in the field reveals that most vaccines used in the freshwater phase of production were administered by injection, while oral booster vaccinations predominated during the seawater phase (25).

Regarding the route of administration, the Chilean Agricultural and Livestock Service (SAG) requires a higher level of efficacy for oral and injectable vaccines than for immersion vaccines (Oral and injectable: RPS_60_ greater than or equal to 70%; Immersion: RPS_60_ greater than or equal to 60%) (28). Thus, it is tacitly assumed that oral and injection vaccines work better than immersion ones, perhaps because dip vaccines fail to capture antigens efficiently (101). Because most *P. salmonis* vaccines are administered *via* intraperitoneal injection, few studies have compared the efficacy of these vaccines with oral vaccines (102, 103). For example, Tobar and colleagues in 2011 demonstrated that an oral vaccination was able to protect fish against a *P. salmonis* challenge when administered either as a primary vaccination or as a booster for an injected vaccine (103). More recently, Sotomayor-Gerding and colleagues shown that an oral vaccine produced an acquired immune response (IgM) similar to the injectable vaccine (102). On the contrary, the most in-depth study of the efficacy of vaccines against *P. salmonis* in the field have not been able to demonstrate a beneficial effect of oral booster vaccinations (29), perhaps because of one of the extrinsic factors discussed above. The main challenge of oral immunization is to protect the antigens from the harsh environments in the gut so they can remain enough time to be absorbed and taken up by immune cells. Thus, the two oral vaccines previously described by encapsulate their antigens before being administered (102, 103). Tobar and and colleagues used a bioadhesive cationic polysaccharide formulation called MicroMatrix™ by Advanced BioNutrition (103), while Sotomayor and colleagues encapsulated their *P. salmonis* antigens in alginate (linear unbranched polysaccharides isolated from brown alga) using the dispersion technique known as aerodynamically assisted jetting system (102). Interestingly, Sotomayor describes that only 3 times more doses of antigens than the injectable vaccine was needed to achieve similar levels of immune response. Finally, the main advantage of oral immunization over injectable vaccination is that it allows mass immunization without causing stress in the fish (101).

The efficacy of vaccines against *P. salmonis* is regularly evaluated under laboratory conditions, however some laboratory evaluations don’t mimic field conditions so it has been suggested that they might overestimate the true vaccine efficacy (72). Cohabitation challenges are known to better mimic the natural route of infection compared to intraperitoneal injection (IP) challenges (104, 105). In fact, cohabitation is the recommended method for evaluating vaccine efficacy in fish according to the European Medicines Agency (106). However, IP is the main method for evaluating vaccines against *P. salmonis* since it induces the highest and fastest mortality (103, 107–109). A strong local response in the peritoneal cavity in IP challenge with *P. salmonis* has been observed (72), producing a large increase in antibodies, IgM-antibody-secreting cells (ASCs), and *P. salmonis*-specific ASCs up to 6 weeks post-infection (72). Thus (72), suggested that this strong local response could lead to an overestimation of vaccine efficacy if fish are IP-challenged a few weeks after vaccination. These results are consistent and explain very well the low efficacy of some experimental and commercial vaccines observed in cohabitation trials despite achieving high amounts of host IGM expression (61, 110–113).

**Figure 8:**
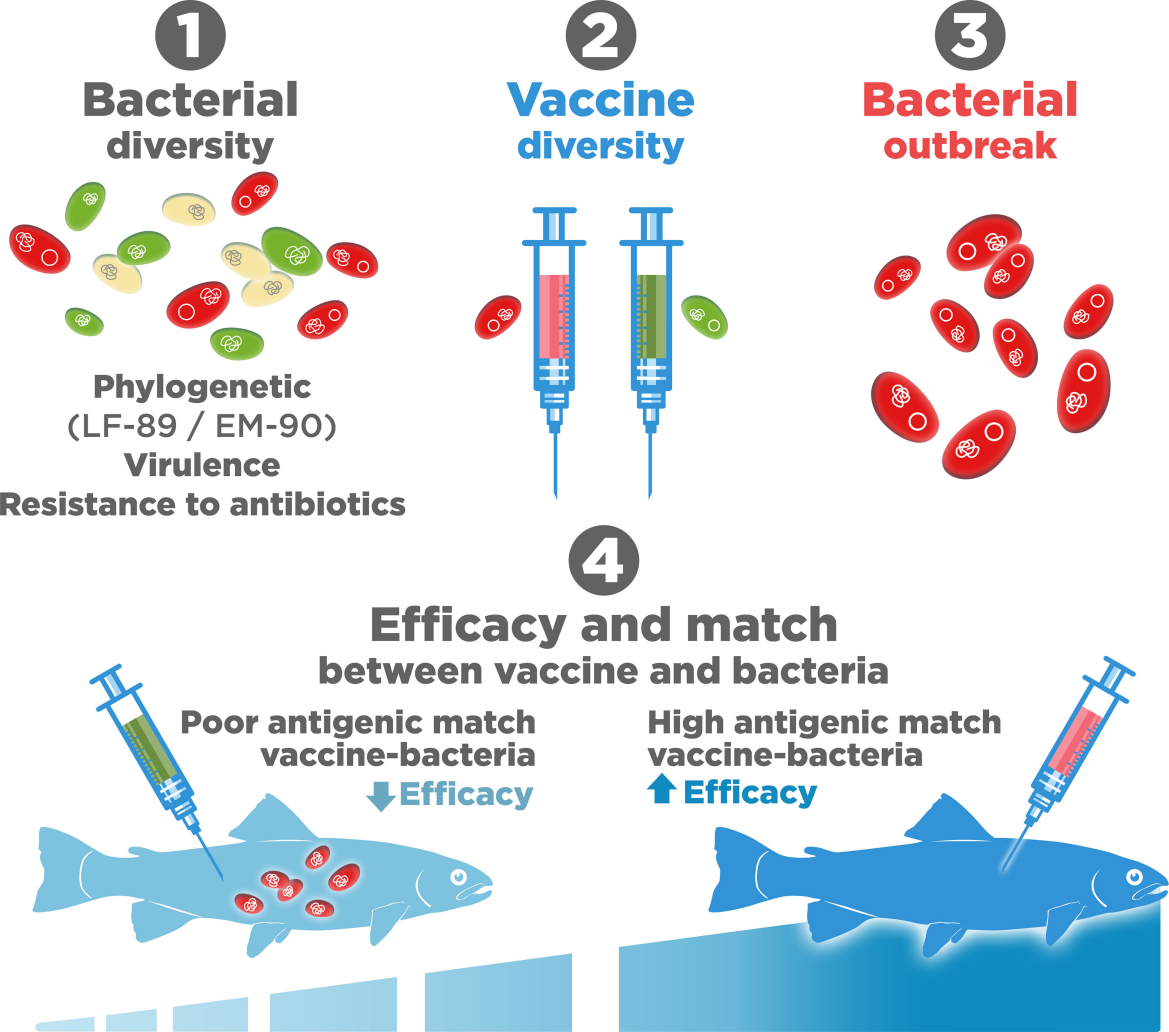
Bacterial diversity and antigenic match with vaccines.

Another important factor, scarcely evaluated, is the antigenic match between the *P. salmonis* strains and the vaccine (**Figure 8**), which could be relevant, since the two most common genogroups present in Chile show major differences in their levels of infectivity and pathogenesis (89, 114). Recently, it has been suggested that some commercial vaccines fail to protect against the two most common genogroups, EM-90 and LF-89 (115). The reason for this failure is widely debated, but perhaps it is because these genogroups should be recognized as separate species as proposed by Schober (2022) (78).

Finally, we cannot rule out problems that could occur in manufacturing vaccines or delivering doses in the field. Manufacturing problems can be associated with incomplete attenuation, while delivery failures may be caused by the interruption of the cold chain or failures in the preparation of the doses. Moreover, the elaboration and production of formalin- or heat-inactivated bacterin vaccines results in low or variable protection against SRS (71). The observed variability could be related to epitope changes caused by inactivation using heat and formalin (71). This could cause a degradation of the antigens and, consequently, limited adaptive immune responses, necessary for the total elimination of the pathogen (14). Although it has been established that bacterin vaccines induce humoral immune responses, the protective mechanism acts through the opsonization of extracellularly replicating pathogens (14, 65), which partially explains the low efficiency observed under field conditions. Finally, it cannot be ruled out that the quality of the vaccination may be impaired when thousands of fish are vaccinated in a short period of time. For example, deviation from the vaccination point may increase mortality and reduce the efficiency of vaccine.

## 4 How to avoid vaccines fail against Piscirickettsiosis

### 4.1 Experimental vaccines: a new approach

Previous studies have shown the feasibility of *P. salmonis* antigens for activating the adaptive immune response; however, commercial vaccines were able to elicit a humoral, but not a cell-mediated, immune response. This point has been addressed regarding the development of novel experimental vaccines with the aim of activating a cellular-mediated response, thereby improving the efficiency under field conditions. Recently, Pontigo and collaborators developed a vaccine prototype that included antigenic proteins from *P. salmonis*, which improved the innate and acquired responses, achieving an RPS of 89.6% three months after inoculation (71). Similarly, studies with recombinant chimeric proteins related to iron metabolism have been conducted (116). Chimeric proteins were used as antigens or reinforcements for commercial vaccines for fish, improving the immune responses in fish infected with more than one pathogen after 400 degree-days post-inoculation (116). Gonzalez-Stegmaier and collaborators postulated that using recombinant flagellin proteins as immunostimulants in conjunction with commercial vaccines against *P. salmonis* could improve the efficacy of the vaccines by promoting a rapid acute inflammatory state, causing an increase in IgM production (117). Another experimental vaccine developed based on *P. salmonis* antigens was tested by Caruffo et al. (14). They used proteoliposomes obtained from the membrane antigens of the intracellular pathogen and managed to obtain an RPS close to 46%, as well as the ability to induce specific anti-*P. salmonis* antibodies (IgM) and an expression profile that suggested cell-mediated immunity (14). Furthermore, other strategies have been designed with the aim of improving vaccine performance. Fuentealba and colleagues engineered a defined culture medium in which to grow *P. salmonis* by increasing the biomass and reducing the amino acids added to the culture medium, which could optimize vaccine production (118). Innovations in vaccine design and formulation are also crucial for the control and inhibition of *P. salmonis’* harmful effects in salmonid cultures.

### 4.2 Autogenous vaccines: Old ideas that seem effective

Autogenous vaccines provide an emergency solution for combating insidious pathogens and has been proposed to control *P. salmonis*. These are vaccines produced from pathogens directly isolated from the affected farms, in which the elaborated vaccines can be implemented at lower frequencies and with more control (119, 120). The benefits of this type of vaccine are its effectiveness against local serotypes of variable pathogens and its ability to be rapidly reformulated compared to regular commercial vaccines (119, 121). Additionally, autogenous vaccines can control outbreaks and decrease the use of antimicrobials (119). Some successful examples of autogenous vaccines against intracellular bacteria have been developed against *Renibacterium salmoninarum* and *Yersinia ruckeri* in salmonids, *Francisella noatunensis* subsp. *orientalis* in *Oreochromis niloticus*, and *Edwardsiella ictaluri* in *Pangasianodon hypophthalmus* (119, 122, 123). Furthermore, some autogenous vaccines have been approved in Chile for use against *Renibacterium salmoninarum* (122). Autogenous vaccines could be a valuable alternative when seeking to reduce the mortality associated with outbreaks of *P. salmonis* while waiting for effective vaccine alternatives to be developed.

### 4.3 New measures to evaluate the efficacy of vaccines

New measures have been proposed to evaluate the efficacy of vaccines, mainly because RPS value may not be a good unit of measure. RPS approach uses the data of the mortalities of two groups challenged with a pathogen, one group being vaccinated and the other unvaccinated (124); the RPS formula therewith calculates the survival attributable to the vaccine (124). However, the disadvantage of RPS is that it does reflect the absolute risk without vaccination, and the number of animals used influences its value. The use of a combination of other parameters such as the relative risk reduction (RRR), absolute risk reduction (ARR), and number necessary to treat (NNT) may afford a better estimation of vaccine efficacy (125, 126). RRR estimates how much the vaccine reduces the risk that exists without vaccination, similar to RPS calculation (125). However, the RRR is calculated by subtracting the relative risk value (RR) from 1. RR is the absolute risk in the treatment (ART) group divided by the absolute risk in the control (ARC) group (ART/ARC). Then, the RRR is the result of 1-RR (126). On the other hand, the ARR is not derived from in an intrinsic property of the vaccine; it is a predictor of the attributable benefits of the vaccination. The result is the interaction between the vaccine and the baseline population risk and is calculated as ARC-ART (127). Finally, NNT is the number of fish that must be treated for one of them to benefit from the vaccine, i.e. to avoid the endpoint of the study. NNT is calculated as the inverse of the ARR, that is, 1/ARR (126). These parameters are currently complementary in the evaluation of vaccine performance and enable a more accurate idea of the efficiency of the vaccine. However, few studies have used the ARR, RRR, or NNT to complement or replace the RPS. An example of *P. salmonis* vaccine evaluation using these parameters is the study by Caruffo and collaborators (14). They evaluated the performance of a proteoliposome-based vaccine against *P. salmonis* with the RPS, ARR, and NNT parameters (14). The results showed an RPS of 46%, an ARR of 36%, and an NNT = 3 (14)—that is, the survival attributable to the vaccine, the reduction in the death risk, and the number of fish needed to be treated to prevent the death of one fish, respectively.

## 5 Conclusion and perspectives

Although vaccines have been considered crucial for the development of salmon farming worldwide (128), they have not been successful against all the pathogens treated, especially intracellular pathogens such as *P. salmonis* (121). The literature summarized here explores the extrinsic and intrinsic factors that impact vaccines’ performance against *Piscirickettsiosis*. Based on the available evidence, to improve the development and the efficacy of vaccines against *P. salmonis* in the field we recommend: a) Do not to perform efficacy evaluations using intraperitoneal injection of pathogens because they generate a generic and short-lived protective immune response, instead challenges of cohabitation or immersion should be used; b) Evaluate the diversity of strains in the field and ensure a good antigenic match with the vaccines; c) Investigate whether host genetic diversity can be used, e.g. through selection, in favor of better and longer response to vaccination; d) To reduce the stressful effects at the cage level, controlling the co-infection of pathogens and avoiding fish overcrowding. To date, we do not know the immunological mechanisms by which the vaccines against *P. salmonis* may or may not generate protection. More studies are required to identify what type of response, cellular or molecular, is required to develop effective vaccines.

## Author contributions

PV-A, DT, and JG-M performed the conceptualization and wrote the first review version. All authors contributed to the article and approved the submitted version.

## Funding

ANID-Chile funded this research study through the project FONDECYT No. 1140772 awarded to JG-M and PC; the Cooperative Research Program Fellowships of OECD (PCI 2015-CONICYT) was awarded to JG-M; and the Chile–Sweden Genomics Project for the Coinfection of Pathogens in Salmonids No. CS2018-7993 was awarded to JG-M. PV-A was supported by two doctoral fellowships from the Universidad Técnica Federico Santa María and ANID N°21220464. ANID-Chile supported D.T. through a postdoctoral fellowship (FONDECYT No. 3210502). C.F. was supported by Pontificia Universidad Católica de Valparaíso and ANID-Chile through two postdoctoral fellowships: Proyecto VRIEA-PUCV Postdoctorado and FONDECYT No. 3170744.

## Acknowledgments

The authors thank Nicol Delgado and Joan Carles Balasch for preparing the figures included in this article.

## Conflict of interest

We declare that JG-M, DT, LM, and PC provided genetic and immunological services to the Chilean salmon industry when this review was written.

The remaining authors declare that the research was conducted in the absence of any commercial or financial relationships that could be construed as a potential conflict of interest.

## Publisher’s note

All claims expressed in this article are solely those of the authors and do not necessarily represent those of their affiliated organizations, or those of the publisher, the editors and the reviewers. Any product that may be evaluated in this article, or claim that may be made by its manufacturer, is not guaranteed or endorsed by the publisher.
