# Supplementary material for: Why vaccines fail against Piscirickettsiosis in farmed salmon and trout and how to avoid it: A review
Source: Front Immunol. 2022 Nov 17;13:1019404. doi: 10.3389/fimmu.2022.1019404 (PMC9714679; doi:10.3389/fimmu.2022.1019404)
Supplement: Supplementary file 1 [file DataSheet_1.docx]

Supplementary Material

Supplementary Table 1.

Use of antibiotics and harvest biomass in Chilean salmon and trout farming between 2007 and 2021.

| Year | Total antibiotics Chilean salmon aquaculture - Tons | Chilean salmon biomass  (Tosn harvested) | Ratio - Grams of antibiotics per Ton harvested |
| --- | --- | --- | --- |
| 2007 | 385,6 | 600.862 | 641,7 |
| 2008 | 325,6 | 630.647 | 516,3 |
| 2009 | 184,5 | 474.174 | 389,1 |
| 2010 | 143,2 | 466.857 | 306,7 |
| 2011 | 206,8 | 649.492 | 318,4 |
| 2012 | 337,9 | 826.949 | 408,6 |
| 2013 | 450,7 | 786.091 | 573,3 |
| 2014 | 563,2 | 955.179 | 589,6 |
| 2015 | 557,2 | 883.102 | 631,0 |
| 2016 | 382,5 | 727.812 | 525,5 |
| 2017 | 393,9 | 855.326 | 460,5 |
| 2018 | 322,7 | 923.900 | 349,3 |
| 2019 | 334,1 | 989.546 | 337,6 |
| 2020 | 379,6 | 1.075.896 | 352,8 |
| 2021 | 463,4 | 985.958 | 470,0 |
| **Mean** | **362,1** | **788786,1** | **458,0** |

Supplementary Table 2.

Provisional registration of commercial vaccines against P*. salmonis* for salmonids in Chile classified by type of vaccine and pathogens. Source: Agricultural and Livestock Service (SAG), 2021. Abbreviations: IPNV: Infectious Pancreatic Necrosis Virus; ISAV: Infectious Salmon Anemia Virus. No vaccine states the duration of immunity (protection) or whether there is any immunogenic incompatibility.

| Registration SAG No. | Type of Vaccine | Pathogens | Name strain | Isolation date | Administration route | Disease prevention | Reduction in mortality, clinical signs, or injuries attributed | Immunity stimulation | Start immunity (UTA) | RPS_60_ (%) |
| --- | --- | --- | --- | --- | --- | --- | --- | --- | --- | --- |
| 1743-BP | Monovalent | *P. salmonis* | Undeclared | Undeclared | Injectable | Yes | Undeclared | Undeclared | Undeclared | Undeclared |
| 1868-BP | Monovalent | *P. salmonis* | PS 2C | Undeclared | Injectable | Yes | Undeclared | Active | Undeclared | Undeclared |
| 2027-BP | Monovalent | *P. salmonis* | PS 2C | Undeclared | Oral | Yes | Undeclared | Active | Undeclared | Undeclared |
| 2136-BP | Monovalent | *P. salmonis* | 238 | Undeclared | Injectable | Yes | Undeclared | Active | Undeclared | Undeclared |
| 2165-BP | Monovalent | *P. salmonis* | Undeclared | Undeclared | Injectable | Yes | Undeclared | Undeclared | Undeclared | 81.5% |
| 2225-BP | Monovalent | *P. salmonis* | 238 | Undeclared | Immersion | Yes | Undeclared | Active | Undeclared | Undeclared |
| 2320-BP | Monovalent | *P. salmonis* | AL 20542 | Undeclared | Injectable | Undeclared | Yes | Active | 456 | Undeclared |
| 1914-BP | Bivalent | *P. salmonis,* IPNV | Undeclared | Undeclared | Injectable | Yes | Undeclared | Active | Undeclared | Undeclared |
| 1956-BP | Bivalent | *P. salmonis,* IPNV | PS 2C | Undeclared | Injectable | Yes | Undeclared | Active | Undeclared | Undeclared |
| 2016-BP | Bivalent | *P. salmonis,* IPNV | Undeclared | Undeclared | Injectable | Undeclared | Yes | Undeclared | 600 | Undeclared |
| 2089-BP | Bivalent | *P. salmonis,* IPNV | AT-01-CHILE-01 | Undeclared | Injectable | Yes | Undeclared | Undeclared | Undeclared | Undeclared |
| 2130-BP | Bivalent | *P. salmonis,* IPNV | AG5,  AG16 | Undeclared | Injectable | Undeclared | Undeclared | Undeclared | Undeclared | Undeclared |
| 2149-BP | Bivalent | *P. salmonis,* ISAV | Undeclared | Undeclared | Oral | Undeclared | Undeclared | Undeclared | Undeclared | Undeclared |
| 2166-BP | Bivalent | *P. salmonis, IPNV* | Undeclared | Undeclared | Injectable | Yes | Undeclared | Undeclared | Undeclared | 84.3 |
| 2217-BP | Bivalent | *P. salmonis,* IPNV | Undeclared | Undeclared | Injectable | Yes | Undeclared | Active | 500 | Undeclared |
| 2237-BP | Bivalent | *P. salmonis,* IPNV | 238 | Undeclared | Injectable | Yes | Undeclared | Undeclared | Undeclared | Undeclared |
| 2448-BP | Bivalent | P. salmonis, IPNV | Undeclared | Undeclared | Injectable | Undeclared | Undeclared | Undeclared | Undeclared | Undeclared |
| 1915-BP | Trivalent | *P. salmonis,* IPNV*,* Vibriosis | Undeclared | Undeclared | Injectable | Yes | Undeclared | Undeclared | Undeclared | Undeclared |
| 1930-BP | Trivalent | *P. salmonis,* IPNV*,* Vibriosis | AL 10015 | Undeclared | Injectable | Yes | Undeclared | Active | 600 | Undeclared |
| 1936-BP | Trivalent | *P. salmonis,* IPNV*,* Vibriosis | PS 2C | Undeclared | Injectable | Yes | Undeclared | Active | Undeclared | Undeclared |
| 2167-BP | Trivalent | *P. salmonis,* IPNV*,* Vibriosis | Undeclared | Undeclared | Injectable | Yes | Undeclared | Undeclared | Undeclared | 80.5 |
| 2233-BP | Trivalent | *P. salmonis*, IPNV, Vibriosis | Undeclared | Undeclared | Injectable | Undeclared | Undeclared | Undeclared | Undeclared | Undeclared |
| 2449-BP | Trivalent | *P. salmonis*, IPNV, Vibriosis | Undeclared | Undeclared | Injectable | Undeclared | Undeclared | Undeclared | Undeclared | Undeclared |
| 1885-BP | Quadrivalent | *P. salmonis,* IPNV*,* Vibriosis*,* Atypical Furunculosis | AL 10015 | Undeclared | Injectable | Undeclared | Yes | Active | 600 | Undeclared |
| 1898-BP | Quadrivalent | *P. salmonis,* IPNV*,* Vibriosis*,* Atypical Furunculosis | Undeclared | Undeclared | Injectable | Yes | Undeclared | Undeclared | Undeclared | Undeclared |
| 2236-BP | Quadrivalent | *P. salmonis,* IPNV*,* Vibriosis*,* Atypical Furunculosis | Undeclared | Undeclared | Injectable | Undeclared | Undeclared | Undeclared | Undeclared | Undeclared |
| 2450-BP | Quadrivalent | *P. salmonis,* IPNV*,* Vibriosis*,* Atypical Furunculosis | Undeclared | Undeclared | Injectable | Undeclared | Undeclared | Undeclared | Undeclared | Undeclared |
| 2061-BP | Quadrivalent | *P. salmonis,* IPNV*,* Vibriosis*,* ISAV | Undeclared | Undeclared | Injectable | Yes | Undeclared | Undeclared | Undeclared | Undeclared |
| 2069-BP | Quadrivalent | *P. salmonis,* IPNV*,* Vibriosis*,* ISAV | PS 2C | Undeclared | Injectable | Yes | Undeclared | Active | Undeclared | Undeclared |
| 2195-BP | Quadrivalent | *P. salmonis,* IPNV*,* Vibriosis*,* ISAV | Undeclared | Undeclared | Injectable | Yes | Undeclared | Undeclared | Undeclared | 87.7 |
| 2074-BP | Pentavalent | *P. salmonis,* IPNV*,* Vibriosis*,* Atypical Furunculosis, ISAV | Al 10005 | Undeclared | Injectable | Yes | Undeclared | Undeclared | 600 | Undeclared |
| 2119-BP | Pentavalent | *P. salmonis,* IPNV*,* Vibriosis*,* Atypical Furunculosis, ISAV | Undeclared | Undeclared | Injectable | Yes | Undeclared | Undeclared | Undeclared | Undeclared |
| 2120-BP | Pentavalent | *P. salmonis,* IPNV*,* Vibriosis*,* Atypical Furunculosis, ISAV | PS 2C | Undeclared | Injectable | Yes | Undeclared | Active | Undeclared | Undeclared |
| 2194-BP | Pentavalent | *P. salmonis,* IPNV*,* Vibriosis*,* Atypical Furunculosis, ISAV | Undeclared | Undeclared | Injectable | Yes | Undeclared | Undeclared | Undeclared | 83.2 |
